# Niche distribution and influence of environmental parameters in marine microbial communities: a systematic review

**DOI:** 10.7717/peerj.1008

**Published:** 2015-06-16

**Authors:** Felipe H. Coutinho, Pedro M. Meirelles, Ana Paula B. Moreira, Rodolfo P. Paranhos, Bas E. Dutilh, Fabiano L. Thompson

**Affiliations:** 1 Universidade Federal do Rio de Janeiro (UFRJ)/Instituto de Biologia (IB), Rio de Janeiro, Brazil; 2 Radboud University Medical Centre, Radboud Institute for Molecular Life Sciences, Centre for Molecular and Biomolecular Informatics (CMBI), Nijmegen, The Netherlands; 3 Universidade Federal do Rio de Janeiro (UFRJ)/COPPE, SAGE, Rio de Janeiro, Brazil; 4 University of Utrecht (UU), Theoretical Biology and Bioinformatics, Utrecht, The Netherlands

**Keywords:** Metagenomics, Community ecology, Species interactions, Microbial ecology, Global ocean

## Abstract

Associations between microorganisms occur extensively throughout Earth’s oceans. Understanding how microbial communities are assembled and how the presence or absence of species is related to that of others are central goals of microbial ecology. Here, we investigate co-occurrence associations between marine prokaryotes by combining 180 new and publicly available metagenomic datasets from different oceans in a large-scale meta-analysis. A co-occurrence network was created by calculating correlation scores between the abundances of microorganisms in metagenomes. A total of 1,906 correlations amongst 297 organisms were detected, segregating them into 11 major groups that occupy distinct ecological niches. Additionally, by analyzing the oceanographic parameters measured for a selected number of sampling sites, we characterized the influence of environmental variables over each of these 11 groups. Clustering organisms into groups of taxa that have similar ecology, allowed the detection of several significant correlations that could not be observed for the taxa individually.

## Introduction

Assembly of microbial communities is believed to be simultaneously regulated by stochastic and deterministic processes (Langenheder & Szekely, 2011; Jeraldo et al., 2012; Stegen et al., 2012). Neutral theory postulates that the composition of biological communities is determined by stochastic processes only. In an extreme version of this theory, all species are considered ecologically equivalent, and their abundances between environments are influenced exclusively by random events of birth, death and dispersion (Jeraldo et al., 2012). In contrast, niche theory is based on the assumption that the species composition of an ecosystem is entirely determined by environmental conditions, a process known as habitat filtering (Dumbrell et al., 2010; Pontarp et al., 2012). This process results in communities of co-existing organisms with largely overlapping ecological niches, meaning that they respond similarly to environmental conditions of their habitats and possibly compete for resources (Ulrich et al., 2009; Maire et al., 2012). In contrast, niche partitioning, allows co-occurring microorganisms to avoid competition by using different strategies to exploit the diversity of resources available at their environment (Macalady et al., 2008).

Co-occurrence patterns between organisms can reveal ecological associations that take place between the members of a community. For example, if two organisms are frequently present together, and absent together, across multiple environments or samples, this can be interpreted as evidence that they occupy similar ecological niches (Horner-Devine et al., 2007; Faust & Raes, 2012). Observing ecological associations among microbes *in situ* represents a much less trivial task than doing so for animals and plants. Therefore, analysis of co-occurrence networks represents an alternative to infer possible associations between microorganisms (Barberan et al., 2012; Eiler, Heinrich & Bertilsson, 2012; Faust & Raes, 2012), and between microorganisms and environmental parameters (Ruan et al., 2006; Gilbert et al., 2012).

Here, we performed a meta-analysis of marine metagenomes from pelagic regions of the oceans around the globe, which includes previously published and new metagenomes from the South Atlantic Ocean, a poorly characterized marine realm. We identified patterns of co-variation between members of the marine microbiome. Our analysis identified hundreds of significant correlations that were used to build a co-occurrence network that sheds light into ecological processes taking place in the global ocean. Clustering the taxa of the network revealed groups of co-occurring prokaryotes that share a similar ecological niche. Next, we describe relationships between these groups and environmental parameters. Our results contribute to a better understanding of the processes that govern community assembly and inter-species co-occurrence patterns in the pelagic oceans, and provide important general insights for the understanding of microbial ecology.

## Methods

### Samples

**Figure 1:**
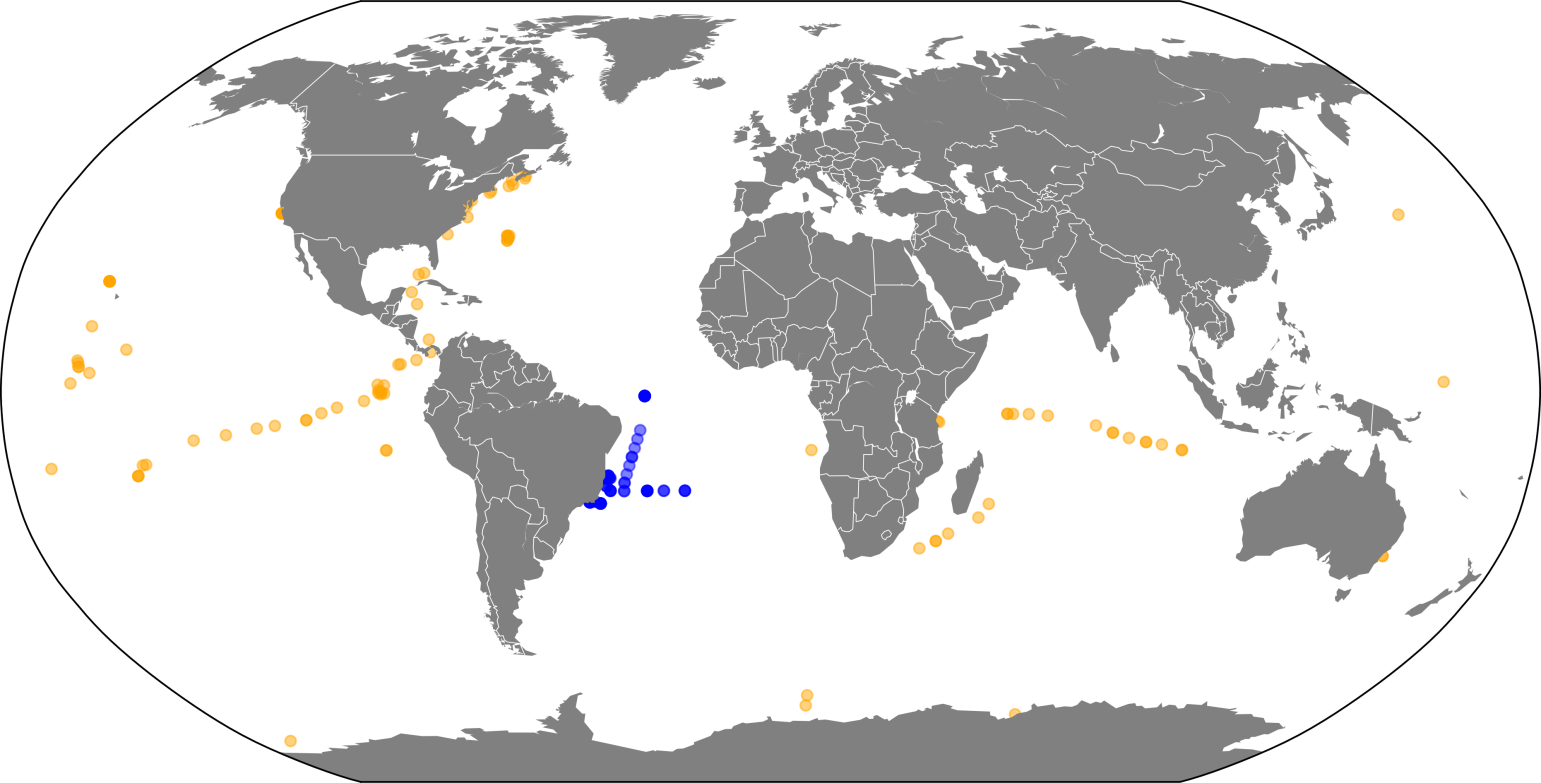
Sample locations. Map of metagenome sampling sites: Blue circles represent metagenomes sampled at the South Atlantic Ocean. Yellow circles represent publicly available metagenomes from other regions of the planet.

A collection of 180 metagenomes were retrieved from MG-RAST (Meyer et al., 2008) (Table S1). Samples covered four major global oceans (Atlantic, Pacific, Indian, and Antarctic) and a broad depth range (0–4,800 m). Sampling sites of each metagenome are illustrated in Fig. 1. Among these samples, 71 metagenomes were obtained from the South Atlantic Ocean. These metagenomes were sampled, processed and analyzed as previously described (Bruce et al., 2012; Alves Jr et al., 2014). The remaining 109 metagenomes were obtained from distinct sites throughout the planet and were publicly available at the MG-RAST server. We chose our dataset aiming to cover a broad range of environmental conditions, allowing for enough variation in microbial abundance to occur between samples so that significant correlations can be detected. Our methodology has been shown to be appropriate to detect associations between microorganisms that can provide insightful information on their ecology (Fuhrman & Steele, 2008; Beman, Steele & Fuhrman, 2011; Steele et al., 2011; Barberan et al., 2012; Eiler, Heinrich & Bertilsson, 2012; Faust & Raes, 2012). The differences in the environmental characteristics of the samples (e.g., location, season, depth) are required so that enough variation exists between samples so that relevant co-occurrence patterns can be detected. If we were to work with samples that were too homogeneous, very little variation would be observed concerning taxon abundances and environmental parameters, impairing the detection of relevant correlations. This broad range of environments provides the variation among samples that is necessary for non-spurious correlations to be detected, among taxa and also between taxa and environmental parameters (Barberan et al., 2012).

Prior to analysis, sequences from the MG-RAST metagenomes were de-replicated and filtered according to Phred score (≥20) and length (≥75 bp). No assembly was performed as to preserve the quantitative information within the metagenomes and to avoid the formation of chimeric sequences. All metagenomes were subjected to the same analysis pipeline. Taxonomic annotation was performed through the MG-RAST server best hit classification. Raw reads were translated in all 6 frames and aligned against Genbank as the reference database through BLAT (Meyer et al., 2008). This database was chosen due to the richness of complete genomes of marine microbes within it, such as those sequenced by the The Gordon and Betty Moore Foundation Marine Microbial Genome Sequencing Project. The cut-off parameters for annotation of a sequencing read were: e-value ≤10^−5^ and sequence identity ≥60%. Raw taxonomic counts were converted to relative abundances by dividing the count of each taxon by the total of annotated reads in each metagenome. A complete list of all 180 metagenomes including their MG-RAST identifiers, total number of reads, average read length, total bases, geographical coordinates, depth and original publication are available as Table S1.

### Physical and chemical parameters

The South Atlantic sampling sites were characterized regarding their water quality conditions, at the time of sampling, by the following methods: Chlorophyll-a analysis was performed following positive pressure filtration of 2 L of seawater. Filters (glass fiber Whatman GF/F) were kept overnight under a solution of 90% acetone at 4 °C for extraction, and analyzed by spectrophotometry or fluorimetry. One liter of water from each sampling site was frozen and stored for further analysis of inorganic nutrients through the following methods: (1) ammonia by indophenol, (2) nitrite by diazotization, (3) nitrate by reduction in Cd–Cu column followed by diazotization, (4) total nitrogen by digestion with potassium persulfate following nitrate determination, (5) orthophosphate by reaction with ascorbic acid, (6) total phosphorous by acid digestion to phosphate, and (7) silicate by reaction with molybdate. All analyses were carried out as previously described (Grasshoff, Kremling & Ehrhardt, 2009; Alves Jr et al., 2014).

### Correlations network

Taxonomic annotations at the genus level were used as we considered any classification at deeper levels (i.e., species or strain) to be unreliable when dealing with the short reads from second generation sequencing technologies, which represent a significant fraction of our samples. Spearman rank correlation scores (*R*) were calculated between the relative abundances of all possible pairwise combinations of taxa. All taxa detected in less than 40% (*n* = 72) of the 180 samples, were excluded from this analysis to prevent sparsely distributed taxa with abundant zero values to yield spuriously high correlation scores. Multiple test correction was performed according to the False Discovery rate (FDR) procedure (Benjamini & Hochberg, 1995). Correlations for which the *p*-value and *q*-value were ≤0.001 and Spearman-*R* score ≤−0.6 or ≥+0.6 were considered significant and plotted as a network through Cytoscape (Saito et al., 2012).

### Node clustering

CFinder (Palla, Barabasi & Vicsek, 2007) was applied to identify clusters of highly connected taxa within the network through the Clique Percolation Method. “Cliques” are defined as groups of nodes (in these case the microbial taxa), which tend to have more connections with each other than with other members of a network. A *k*-step of 3 was chosen for this analysis so that cliques formed by three organisms or more could be identified. Our goal was to identify groups formed by taxa connected by significant positive correlations, therefore negative correlations were not considered by the Clique Percolation Method. Cliques were numbered according to the abundance of nodes assigned to each of them. Henceforth, these cliques will be referred to as “groups”.

### Network consistency

The consistency of the correlations in the network was assessed through a sub-sampling strategy. One hundred new networks were calculated, each from a random sub-sample of 162 out of the 180 metagenomes. Next, the original and new networks were compared and we measured how often each one of the correlations from the original network were also detected in the new networks. The consistency of the groups identified through CPM was assessed by applying the algorithm over the 100 new networks and measuring how often pairs of taxa that were assigned to the same group in the original network also clustered together by CPM in the new networks.

To account for occurrences of spurious correlations between genera due to the compositional (i.e., percentages) nature of our data, correlation scores of the original network were compared against those obtained through SparCC (Friedman & Alm, 2012). This tool was developed to calculate correlations between microbial abundances while eliminating errors that may emerge due to the use of compositional data. SparCC was run using default parameters.

### Correlations between groups and environmental parameters

We addressed the influence of habitat variables over the groups identified by CFinder. For that end, Spearman rank correlation scores were calculated between the relative abundances of the groups and environmental parameters measured for the South Atlantic Ocean samples (*n* = 71, Table S2). Group abundances were calculated as the sum of the relative abundances of all of its members. The environmental parameters used for this analysis were: total nitrogen, nitrite, nitrate, ammonia, total phosphorus, orthophosphate, chlorophyll a, silicate, temperature, depth, latitude and longitude. To identify associations between groups, Spearman correlation scores were calculated for all the possible pairwise combinations of groups. This step of the analysis encompassed all metagenomes (*n* = 180). In both cases, only correlations which yielded a *p*-value ≤0.01 and *q*-value < 0.05 were considered significant.

## Results

The taxonomic composition of 180 marine metagenomes was used to build a network of correlations between genera of microorganisms. Next, the members of this network were clustered in groups based on the significant correlations between them. Correlation scores were measured between pairwise combinations of these groups and between the groups and environmental parameters of the South Atlantic sampling sites.

### Network parameters

**Figure 2:**
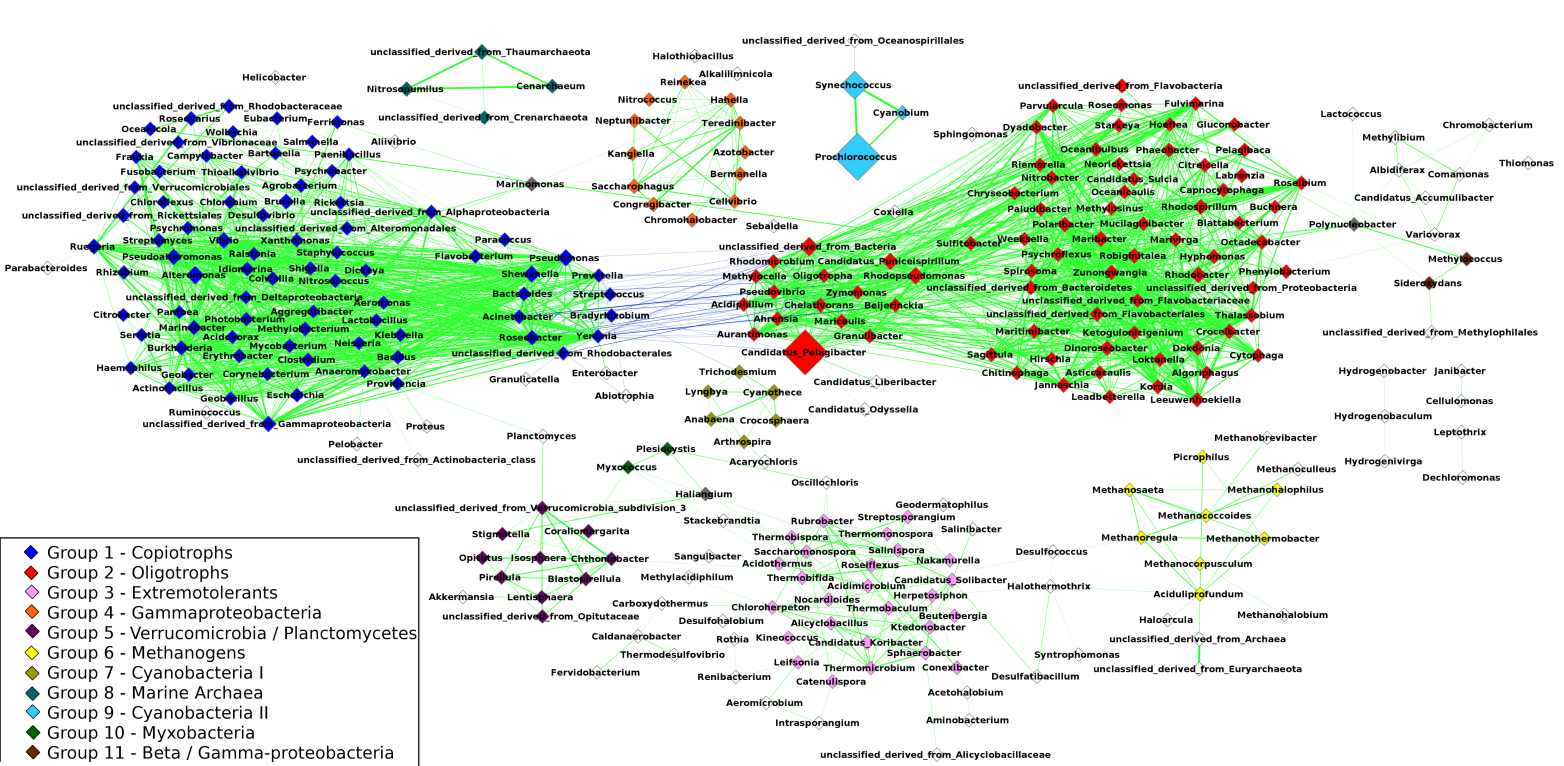
Correlations network: the 1,906 edges linking 297 nodes represent significant correlations between the relative abundances of the connected taxa. Positive correlations are showcased in green while negative ones are in blue. The width of the lines is proportional to the module of Spearman’s *R* of each correlation. Node size represents the average abundance of the taxa across the 180 metagenomes. Nodes are color-coded according to the group to which they were assigned through the Clique Percolation Method. Nodes not assigned to any group are colored in white and nodes assigned to more than a single group (Polynucleobacter, Marinomonas and Haliangium) are colored in gray. For clarity, members of Groups 1 and 2 that are connected by negative correlations are displayed separately from the remaining taxa of their respective groups. *Pelagibacter*, *Prochlorococcus* and *Synechococcus* showed the highest average abundance. Positive correlations dominate the network. The majority of negative correlations were observed between members of groups 1 and 2, between classical examples of oligotrophs and copiotrophs (e.g., *Pelagibacter/Yersinia*). Groups 8 and 9 are isolated, while the remaining groups have at least one edge linking them to other nodes in the network. Strong positive correlations were observed between the members of groups 8 and 9 (e.g., *Synechococcus/Prochlorococcus* and *Cenaracheum/Nitrosopumilus*).

The resulting network was composed of 297 taxa (nodes) and 1,906 correlations (edges) (Fig. 2), of which 1,863 were positive correlations (mean *R* = 0.68 ± 0.06) and 43 were negative correlations (mean *R* = − 0.62 ± 0.02). The network had a clustering coefficient (tendency of nodes to cluster together) of 0.54 and density (the number of edges in the network divided by the possible maximum) of 0.04. The average node degree (number of connections of a node) was 5.3 ± 8.04. Using the Clique Percolation Method, we identified eleven groups of co-occurring microbial taxa, containing between three and eighty members. Of the 297 microbial taxa, 229 were assigned to a single group, while 65 were assigned to none. Three genera where assigned to more than a single group: *Marinomonas* (present in two groups), *Polynucleobacter* (two groups) and *Haliangium* (three groups). The composition of these groups is described in Table S3.

Out of the 1,906 correlations in the original network 1,843 (98%) were consistent in at least half of the sub-sampled networks. Groups assignments also yielded high consistency, 99% of the pairwise group assignments of the original network were consistent across at least 50% of the sub-sampled networks. Comparison between networks calculated through Spearman correlations and SparCC, revealed that 98,7% of all correlations detected in the first were also present in the latter (module o *R* > 0.3), indicating that correlations of the original network are not spurious due to the compositional nature of the data. The difference in correlation scores between the networks are likely the effect of non-linear associations between variables, that cannot be captured by SparCC.

### Composition of groups identified through the Clique Percolation Method

Group 1 harbored 80 genera distributed among seven bacterial phyla (*Firmicutes, Chloroflexi, Fusobacteria, Planctomycetes, Proteobacteria, Bacteroidetes, and Actinobacteria*), this group had more members than any other and also harbored the highest number of phyla. Many of the members of Group 1 are typical genera of heterotrophic aquatic bacteria (e.g., *Vibrio* and *Shewanella*). Group 2, formed by 78 members, was dominated by genera of *Alphaproteobacteria* (e.g., *Puniceispirillum* and *Rhodobacter*) and *Bacteroidetes (e.g., Chryseobacterium* and *Cytophaga)*, this group also harbored the ubiquitous *Pelagibacter* genus. Most of the members of Group 3 were *Actinobacteria* or *Chloroflexi*, but other phyla (e.g., *Acidobacteria, Firmicutes, Proteobacteria* and *Chlorobi*) were also represented in this group. Group 4 was formed by 13 genera of *Gammaproteobacteria*, from the orders *Pseudomonadales, Alteromonadales, Chromatiales and Oceanospirillales*. The majority of genera assigned to Group 5 were either *Verrucomicrobia* or *Planctomycetes*, with only three exceptions: *Haliangium, Stigmatella* and *Lentisphaera*. Group 6 was formed by genera of *Euryarchaeota*, which included several methanogenic organisms (e.g., *Methanothermobacter*). Group 7 was formed by genera of photosynthetic organisms of the phylum *Cyanobacteria* (e.g., *Anabaena, Cyanothece* and *Lyngbya*). Group 8 was composed entirely of *Archaea*, including the genera *Cenarchaeum* and *Nitrosopumilus* as well as unclassified organisms from the phyla *Thaumarchaeota* and *Crenarchaeota*. Group 9 was also composed entirely of *Cyanobacteria*. The photosynthesizers *Prochlorococcus* and *Synechococcus*, both among the most abundant organisms in the analyzed metagenomes, were assigned to this group, along with *Cyanobium*. Group 10 was formed by three genera of *Myxobacteria*: *Myxococcus*, *Plesiocystis and Haliangium*, the latter was also a member of groups 4 and 5. Only three taxa are part of Group 11, all *Proteobacteria*: *Polynucleobacter*, *Methylococcus* and *Sideroxydans*.

### Correlations between groups and environmental parameters

**Figure 3:**
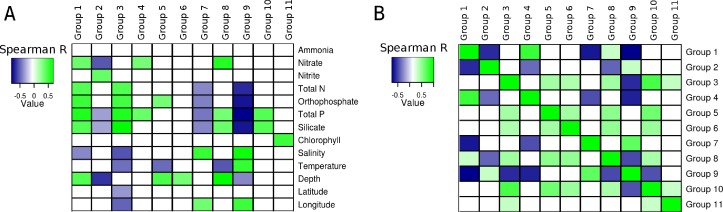
Correlations between groups and environmental parameters. Heatmap of correlation scores: (A) Correlations calculated between group abundances and environmental parameters. (B) Correlations calculated between group abundances. Positive correlations are showcased in green while negative ones are in blue. Non-significant correlations (*p* > 0.01 or *q* > 0.05) are shown as white squares.

Correlations calculated between environmental parameters, of the South Atlantic Ocean and the relative abundance of the groups of microorganisms produced unique patterns for each group (Fig. 3A). With the exception of ammonia, all variables produced at least one significant correlation with at least one group. Total phosphorus produced significant correlations with eight groups, more than any of the other variable. The pattern of correlations detected between silicate and seven of the groups was similar to that observed for total phosphorus, but silicate showed no significant correlation with Group 4. The third variable with most significant correlations was depth (5 groups). The variables that produced least significant correlations were Chlorophyll a (1), latitude (1) and Ammonia (0).

The abundance of Group 1, which harbored many heterotrophic and pathogenic organisms, showed positive correlations with depth and several nutrients (i.e., orthophosphate, total phosphorus, silicate, nitrate and total nitrogen), also, a negative correlation was detected between salinity and the abundance of this group. The abundance of Group 2 (mainly *Alphaproteobacteria* and *Bacteroidetes*) was negatively correlated with three nutrients (nitrate, total phosphorus and silicate). Additionally, a positive correlation with nitrite was observed for this group. Significant positive correlations were obtained between the abundance of Group 3, composed of many extremophilic organisms, and total nitrogen, total phosphorus, orthophosphate and silicate. This same group had negative correlations with salinity, temperature, latitude and longitude. Positive correlations were detected between Group 4 (all *Gammaproteobacteria*) and both nitrate and total phosphorus. The abundance of group 5 showed positive correlations with orthophosphate and depth, and a negative one with temperature. Group 6, composed mainly of methanogenic *Archaea*, had a positive correlation with depth, no other significant correlations were detected between the abundance of these organisms and the other environmental variables. Group 7, composed of *Cyanobacteria*, showed negative correlations with total nitrogen, orthophosphate, total phosphorus and silicate. Positive correlations between salinity and longitude were also observed for Group 7. Positive correlations were detected between Group 8 and nitrate, total phosphorus, silicate and depth, and a negative correlation with temperature. Group 9, also formed by *Cyanobacteria*, showed negative correlations with total nitrogen, orthophosphate, total phosphorus, silicate and depth, positive correlations with salinity, temperature and longitude were also observed. Group 10 showed positive correlations with total phosphorus and silicate. A positive correlation between Chlorophyll and Group 11 was observed, no other significant correlations were observed for this group or this environmental parameter.

Several relationships emerged from correlating the relative abundances of the eleven groups between each other (Fig. 3B). Both positive and negative, significant correlations were detected and each group presented a unique pattern of correlations with the others.

## Discussion

### Assessing technical heterogeneity biases

We opted for using samples derived from a broad spatial range (Fig. 1) so that we could identify microbial occurrence patterns that are applicable to the entirety of the oceans. Ideally, all of our samples would have been processed with the exact same protocols (e.g., for water filtering, DNA extraction and sequencing). Yet, restricting our dataset to one that fits these criteria would result in a very small number of samples, drastically impairing both the power of our approach and the relevance of our results, which emerge from the use of a large number of samples from a broad range of environments. The use of different sequencing technologies can indeed yield slightly different results for taxonomic composition. Nevertheless, these differences were shown to be of very little impact for the overall patterns of community composition (Danhorn, Young & Delong, 2012; Luo et al., 2012; Solonenko et al., 2013). This is in agreement with previous studies which have shown that despite the potential biases that may be introduced by these methodologies, metagenomes carry a strong taxonomic and functional signal which is not surpassed by sample preparation biases (Dinsdale et al., 2008; Willner, Thurber & Rohwer, 2009; Faust et al., 2012).

We addressed the potential biases in metagenomes that could emerge from different sample preparation strategies in different ways. First, we assessed to what extent samples are grouped according to the laboratory by which they were processed. To do this, Euclidean distances were calculated between metagenomes based on their genera composition. These distances were used as input for Principal Coordinates Analysis (PCoA). In a scenario where the taxonomic composition of a metagenome is strongly determined by sample processing, metagenomes are expected to cluster tightly by laboratory. This pattern is not present among our samples (Fig. S1).

Second, we evaluated which of the 297 taxa of the network are over or under-represented in the samples from one laboratory, when compared to the samples from the remaining laboratories, by using the Mann–Whitney test, with multiple testing correction through the FDR. We assume that potentially biased taxa are those that are significantly over or under-represented on samples of a single laboratory. However, if a taxon is over or under-represented across multiple laboratories, it is more likely that this is a true biological signal of the samples analyzed by those projects, rather than a bias emerging from different sample processing methodologies. According to these criteria, only 14 taxa could be potentially biased (*q* value < 0.05, see Table S4 for the full list of taxa, the laboratories in which they are enriched and the groups to which they were assigned within the network according to CFinder). It is not possible to determine if this pattern emerges from a true biological signal within the samples, or if they are the result of sample preparation methodologies. Nevertheless, if the case is the latter this is likely to be over very little influence in the overall results, considering that only a very small fraction of all the taxa in the network fall within this category (<5%).

Third, to further explore issues that might arise from sample preparation, we recalculated a correlations network using only the 71 samples from the South Atlantic ocean sampled by Thompson et al. (see Table S1). These represent the largest consistent group regarding sample preparation strategies. We then compared the correlation scores from the global network (180 metagenomes) to those obtained using only the south Atlantic samples (71 metagenomes, see Table S5). The average absolute Spearman-*R* values of these two networks are respectively 0.67 ± 0.06 and 0.37 ± 0.21 and a significant correlation exists between these values (Pearson R 0.37, *p*-value <2.2^−16^), providing yet another evidence that the 1,906 correlations from the global network are consistent within a homogeneous dataset regarding sample preparation methods and do not result from sample preparation bias.

### Groups are formed by organisms with shared ecological niches

Several genera assigned to Group 1 are copiotrophic bacteria (i.e., thrive in nutrient rich conditions) such as *Vibrio, Pseudomonas, Escherischia* and *Clostridium*. As a consequence of their nutritional demands, these genera are frequently abundant in eutrophic waters (Gilbert et al., 2010; Gregoracci et al., 2012), in which nutrient concentrations are high. In contrast, many of the genera affiliated to Group 2 are oligotrophic bacteria, characteristic of aquatic environments where nutrients are scarce. The extremely abundant genus *Pelagibacter*, which possesses several adaptations to thrive in nutritionally poor waters (Carini et al., 2013; Tripp, 2013) was assigned to this group, along with other genera adapted to live at nutrient deprived environments, such as the chemolitoautotrophic *Oligotropha* (Paul et al., 2008) and the heterotrophic *Maricaulis* (Abraham et al., 1999). Therefore, trophic strategies appear to be the unifying trait of the members of these two groups.

Many of the members of Group 3 are capable of surviving in extreme habitats, such as *Thermomicrobium*, capable of growing in elevated temperatures, and *Acidothermus*, capable of tolerating both low pH and high temperatures (Mohagheghi et al., 1986; Wu et al., 2009). These organisms were detected in metagenomes from samples retrieved at non-extreme environments, and encompassed several phyla. Despite their phylogenetic distance and differences regarding their adaptations to thrive in extreme environments, these genera of extremotolerants may share similar habitat preferences at mesophilic waters. Shared niche can also explain the correlations occurring within Group 6, which is dominated by genera of methanogenic *Archaea*. Co-variation in the abundance of these organisms is expected since methanogenesis usually takes place in very specific environments: rich in organic matter and poorly oxygenated (Peng et al., 2008; Angel, Matthies & Conrad, 2011). Even though we applied a highly conservative e-value cutoff (≤1 × 10^−5^) it is also possible that some of the sequences received incorrect taxonomic assignments, which could explain the presence of extremophilic organisms at mesophilic environments.

The highly abundant genera *Prochlorococcus* and *Synechococcus* were assigned to Group 9, along with *Cyanobium*. Meanwhile the much less abundant genera of *Cyanobacteria* (e.g., *Anabaena* and *Cyanothece*) were all assigned to Group 7. No significant correlations were detected between any of the members of these two groups. Differences in how these two groups make use of the resources available at the marine ecosystem could be responsible for the increased ubiquity and abundance of *Prochlorococcus* and *Synechococcus* among Earth’s oceans, traits which are not shared with the *Cyanobacteria* of Group 7. The success of *Prochlorococcus* sp., especially in oligotrophic waters, has been attributed to reduced genome and cell sizes, as well as high rates of nutrient uptake (Zubkov et al., 2003; Partensky & Garczarek, 2010). Distinctive traits of bacteria from Group 7 include the formation of filamentous colonies in the water column (Rice, Mazur & Haselkorn, 1982; Sanudo-Wilhelmy et al., 2001), blooms (Albert et al., 2005), and also the diazotrophic metabolism present in several of its members (Reddy et al., 1993; Omoregie et al., 2004; Bergman et al., 2013).

Based on these observations, we may conclude that the groups identified in the network are formed by organisms which occupy similar ecological niches (Chaffron et al., 2010; Freilich et al., 2010; Faust et al., 2012). Positive correlations could also be the result of collaborative associations (i.e., mutualism). Our data does not allow us to differentiate positive correlations emerging from sharing of an ecological niche from mutualistic associations (Chaffron et al., 2010; Faust et al., 2012). Although these associations may occur, previous studies have also concluded that positive correlations detected at microbial correlation networks are representative of niche overlap rather than cooperative associations and consider this to be a more parsimonious explanation for the occurrence of these correlations (Foster & Bell, 2012; Levy & Borenstein, 2013).

Previous analysis of microbial networks have reported that phylogenetically related organisms co-occur with each other more than expected by chance, as consequence of sharing a niche (Chaffron et al., 2010; Barberan et al., 2012). Some of the groups identified consisted of phylogenetically related taxa (e.g., groups 4, 6, and 10). However we also observed groups composed of distantly related organisms (e.g., groups 1, 2 and 3). Moreover, closely related organisms sometimes occurred in different groups (e.g., *Cyanobacteria* divided between groups 7 and 9). These patterns suggest that group composition was determined by niche distribution, rather than by phylogenetic relatedness.

It is important to take some matters into consideration when interpreting the results of the network. Organisms that were assigned to the same group do not necessarily occupy identical ecological niches. Instead, organisms of the same group have a higher degree of niche similarity between themselves when compared to other taxa of the network. *Synechoccoccus* and *Prochlorococcus* can be taken as an example of this pattern. These genera were grouped together by the Clique Percolation Method, the two taxa have well defined differences in their niche preferences, with regard to their geographic and temporal distributions, light harvesting apparatus and nutrient acquisition machinery (Partensky, Blanchot & Vaulot, 1999; Scanlan et al., 2009). Despite those differences, the two groups of picocyanobacteria have many similarities in central aspects of their physiology, such as the photosynthetic metabolism, reduced cell and genome sizes, and carbon concentration mechanisms. These similarities may be the more important aspects that regulate their response to environmental conditions, thus giving rise to the strong positive correlations observed between their abundances. In addition, the taxonomy of *Synechoccoccus* and *Prochlorococcus* has not been fully elucidated, thus erroneous taxonomic assignments may have contributed for the correlations between the groups.

Also, it is likely that some of the organisms detected in the metagenomes are dormant or inactive. At this state, this organisms are not responding to the fluctuations of the environmental parameters taking place at their habitat. Therefore, no covariation should be expected to occur between these organisms and the active ones. Therefore, no false-positives are expected to arise due to the occurrence of inactive organisms.

### Niche segregation in marine microbial communities

Each of the groups identified by the Clique Percolation Method represent genera that share a similar ecological niche. This means that our analysis detected 11 groups of prokaryotes with distinct ecologies. This is a conservative number since the heterogeneity and extension of the oceans contribute to a much wider diversity of niches to be occupied. Additionally, the number of groups detected will depend on methodological parameters including the *k*-step chosen for the Clique Percolation Method (*k* = 3), the cutoff established for the Spearman correlations scores (≥0.6 or ≤−0.6) and the minimum ubiquity of the taxa among the 180 samples (40%). Nevertheless, the detected correlations and group compositions were consistent in the sub-sampled networks. Finally, since the very rare organisms (i.e., detected in less than 40% of all metagenomes) were excluded from the network it is possible that the groups formed by them were disregarded as well.

Studies based on genomic analysis have shown that small variations in protein encoding genes may lead organisms that belong to the same genus, or even the same species to occupy different niches (Rocap et al., 2003; Kashtan et al., 2014). Species, and strains can have unique traits (e.g., strategies for assimilation of organic compounds and spatial distribution) that set them apart from the other members of their genus (Johnson et al., 2006; Del Carmen Muñoz-Marín et al., 2013; Thompson et al., 2013). These differences could further segregate them into sub-divisions of the 11 groups identified through our network. Unfortunately, due to their length, metagenomic reads generated by second generation sequencing technologies cannot produce reliable annotations at taxonomic levels deeper than genus.

### Influence of environmental parameters

Many associations were detected between the groups and environmental parameters. Yet, none of them yielded perfect correlation scores with the abundance the microbial groups. This is expected considering that the abundance of these groups, in the environment, is regulated by many variables simultaneously. Therefore, it is unlikely that a single measured physical or chemical parameter can adequately explain the abundance of a group across all the samples. Nevertheless, the pattern of significant correlations detected between group abundances and environmental parameters provides insights into how the members of these groups are influenced by environmental variables of their habitat and what niche is occupied by them.

Positive correlations were detected between several nutrients (i.e., nitrate, total nitrogen, orthophosphate, total phosphorus and silicate) and the abundance of Group 1 (Copiotrophs). Meanwhile, negative correlations where detected between Group 2 (Oligotrophs) and nitrate, silicate and total phosphorus (Fig. 3A). This pattern shows that members of Group 1 are more abundant in waters that are rich in these nutrients, while Group 2 is more abundant in regions of the ocean deprived of them. This is corroborated by our observation that an increase in the abundance of one group is accompanied by a reduction in the abundance of the other, leading to significant anti-correlations between the abundances of these organisms, which were observed when comparing the abundances of the genera individually (Fig. 2) and of the whole groups (Fig. 3B).

Groups 7 and 9, composed of *Cyanobacteria*, both showed negative correlations with total nitrogen, orthophosphate, total phosphorus, and silicate. These results are in accordance with genomic analyses of *Synechococcus* sp. and *Prochlorococcus* sp., members of Group 9, which possess several features that contribute to the success of these organisms in oligotrophic conditions (Lauro et al., 2009).

### Clustering unveils the ecology of marine microorganisms

The associations between group abundances and the physicochemical environmental parameters (Fig. 3A) provide insights regarding the ecological roles and niche preferences of the identified groups. Interestingly, such correlations were not significant when these same habitat variables were compared with the abundance of the individual genera that are part of these groups. We suggest that clustering organisms based on co-occurrence is a useful and necessary tool to reveal elusive associations between microorganisms and habitat variables.

Considering the evidence that the organisms from the same group occupy similar niches, it is possible that they are ecologically redundant (i.e., they contribute with the same, or at least similar, roles for ecosystem stability). We propose that each of the groups identified in the network contributes to ecosystem stability by performing a distinct ecological role at the marine environment. Members of a microbial community eventually decline as a result of processes such as phage predation, grazing or a drastic change in environmental conditions. Withering organisms can be replaced by their ecological equivalent, which possesses the necessary features to fill the niche left unoccupied (Gifford et al., 2012). This cycling of species would preserve the ecological roles that are necessary to sustain an ecosystem, as described by the insurance hypothesis (Yachi & Loreau, 1999; Allison & Martiny, 2008). In that case, the influence of environmental parameters would act upon all the members of a niche and not over the individual taxa (as these are redundant and interchangeable), which could explain why these correlations could only be detected at the group level.

## Conclusions

The correlations network proved to be a valuable tool to disclose shared ecological niches in the global ocean microbiome. Habitat filtering and niche segregation may be considered important factors controlling the taxonomic composition of microbial communities from different locations of the global ocean. The data presented here provides insights into the ecological processes that structure marine microbial communities and show that clustering organisms into ecologically cohesive groups may reveal elusive associations between microbes and habitat variables. The advancement of technologies that allow microbial communities to be studied, *in situ* and at higher spatial resolution (i.e., at the micro, rather than the macro-scale) will help broaden the scope of the analysis presented here, allowing for deeper understanding of the ecology of marine microbial communities.

## Supplemental Information

10.7717/peerj.1008/supp-1 Supplemental Information 1 Prisma checklist Description of meta-analysis

10.7717/peerj.1008/supp-2 Supplemental Information 2 Prisma flow diagram Flow diagram of sample selection for meta-analysis.

10.7717/peerj.1008/supp-3 Figure S1 PCoA of 180 metagenomic samples Principal coordinate analysis of the 180 metagenomes based on distances of genera composition using the Euclidean method. Samples are colour coded according to the laboratory by which they were processed.

10.7717/peerj.1008/supp-4 Table S1 Sample metadata Information regarding the metagenomes used in this study.

10.7717/peerj.1008/supp-5 Table S2 Environmental information of the Brazilian coast sampling sites Measurements of environmental variables at the sampling sites of the 71 marine metagenomes from the brazilian coast analyzed.

10.7717/peerj.1008/supp-6 Table S3 Group composition Distribution of taxa across the 11 groups identified through the Clique Percolation Method.

10.7717/peerj.1008/supp-7 Table S4 Potentially biased taxa List of taxa that are over or under represented within the samples of a single laboratory.

10.7717/peerj.1008/supp-8 Table S5 Network comparison Comparisson between the global (180 metagenomes) and south Atlantic (71 metagenomes) networks based on ubiquity of taxa, Spearman *R*, *p*-value and *q*-value.

## Additional Information and Declarations

### DNA Deposition

The following information was supplied regarding the deposition of DNA sequences: MG-RAST 4464153.3 4464162.3 4464167.3 4464168.3 4464169.3 4464170.3 4464171.3 4464173.3 4465773.3 4465774.3 4465775.3 4484652.3 4484653.3 4484654.3 4484655.3 4484656.3 4484657.3 4487641.3 4487642.3 4487643.3 4487644.3 4487645.3 4487646.3.
