# Supplementary material for: Niche distribution and influence of environmental parameters in marine microbial communities: a systematic review
Source: PeerJ. 2015 Jun 16;3:e1008. doi: 10.7717/peerj.1008 (PMC4476133; doi:10.7717/peerj.1008)
Supplement: Supplemental Information 2 — Flow diagram of sample selection for meta-analysis. [file peerj-03-1008-s002.doc]

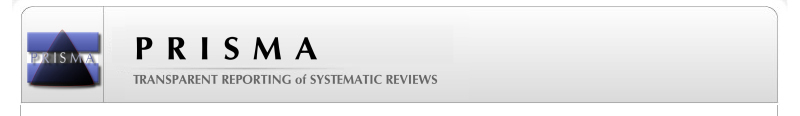
**PRISMA 2009 Flow Diagram**

**Screening**

**Included**

**Eligibility**

**Identification**

Records identified through database searching
(n = 180 )

Additional records identified through other sources
(n = 0)

Records after duplicates removed
(n = 180)

Records screened
(n = 180 )

Records excluded
(n = 0)

Full-text articles assessed for eligibility
(n = 180)

Full-text articles excluded, with reasons
(n = 0 )

Studies included in qualitative synthesis
(n = 180 )

Studies included in quantitative synthesis (meta-analysis)
(n = 180)
