# Supplementary figures and images for: Niche distribution and influence of environmental parameters in marine microbial communities: a systematic review
Source: PeerJ. 2015 Jun 16;3:e1008. doi: 10.7717/peerj.1008 (PMC4476133; doi:10.7717/peerj.1008)

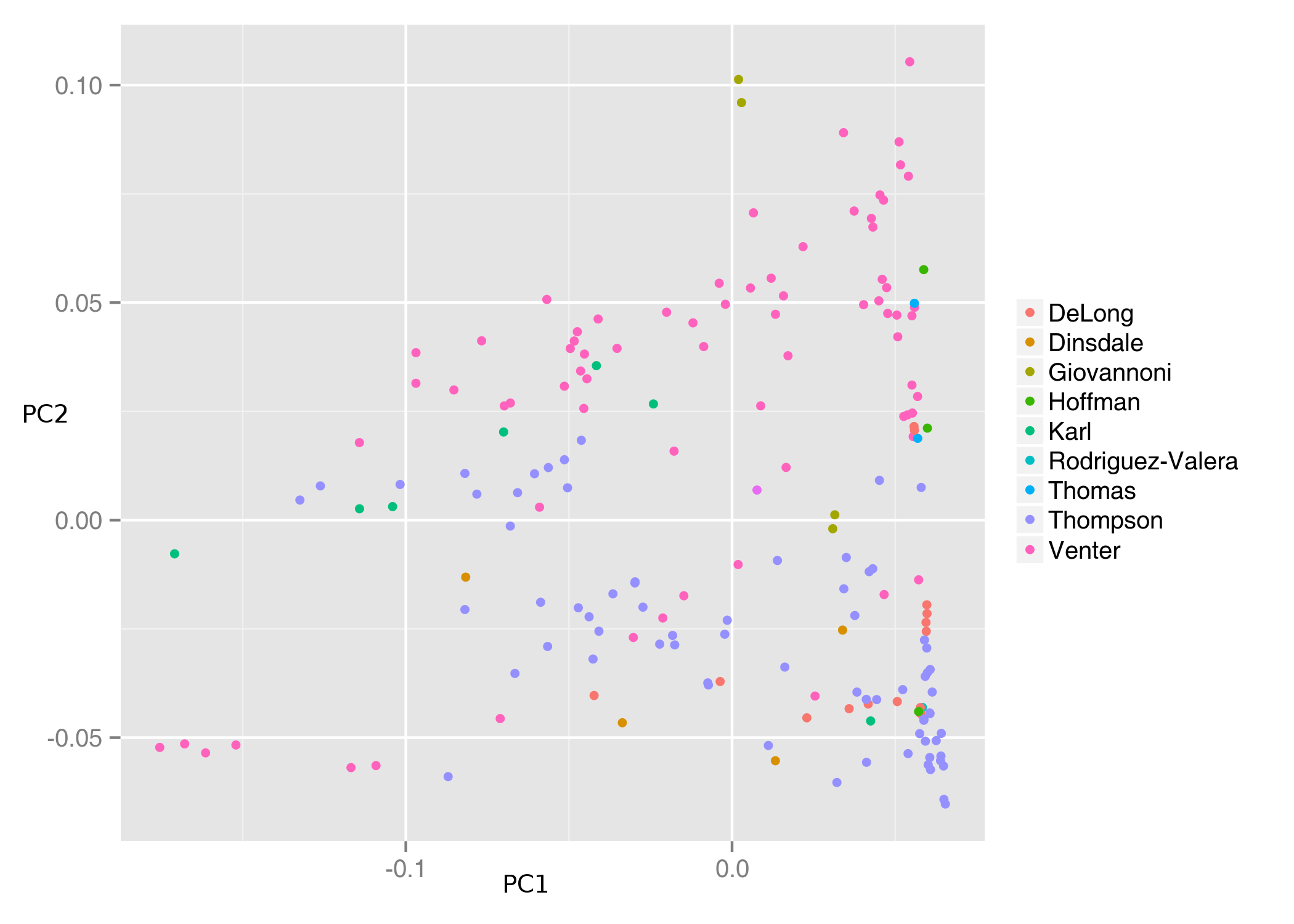

Supplement: Figure S1 — Principal coordinate analysis of the 180 metagenomes based on distances of genera composition using the Euclidean method. Samples are colour coded according to the laboratory by which they were processed. [file peerj-03-1008-s003.png]
